# Tonsil Infections

**Published:** 1919

**Authors:** 


***Tonsil Infections***. By H. J. Nichols and J. H. Bryan. *Journal A. M. A.*, Nov. 30, 1918.

The authors have studied the hemolytic streptococcus infections and are convinced that the tonsils play a part that should be emphasized. Swab cultures were made from various portions of the air passages, and the results pointed strongly to the tonsils as the foci of infection. Additional evidences as to their importance in carriers were found in the results of throat cultures taken after tonsillectomy. The removal of diseased tonsil is, in their opinion, a necessity in carriers for the good of others, and they summarize their conclusions as follows :

1. The tonsils are the principal foci of infection in throat carriers of streptococcus hemolyticus. (*a*) Cultures taken from different parts of the nose, mouth and throat show more streptococci in the tonsils than elsewhere. (*b*) Cultures from excised tonsils show streptococci in 75 per cent. of cases. (*c*) Crypt cultures show a higher percentage of positive results than surface cultures. (*d*) Excision of the tonsils renders the throat negative in nearly all cases.

2. The streptococci isolated from tonsils and throats show no cultural difference on sugar mediums from those obtained from empyema fluids.

3. Excision is the only radical method of curing carriers of infection. (*a*) Dobell's solution, dichloramin -T, and hot alcohol had little better effect than no treatment. (*b*) Silver nitrate, 25 per cent., gave better results in negative throat cultures, but the crypts frequently remained positive.

4. The presence of hemolytic streptococci in pathologic tonsils is an additional reason for their removal.

